# High-performance low-velocity turbulent flow piezoelectric energy harvester using porous Ba_0.85_Ca_0.15_Zr_0.1_Ti_0.9_O_3_ (BCZT) ceramics

**DOI:** 10.1016/j.isci.2025.113881

**Published:** 2025-10-27

**Authors:** Zihe Li, James Roscow, Hamideh Khanbareh, Ruxue Yang, Zhengyang Huo, Geoff Haswell, Min Pan, John Taylor, Chris Bowen

**Affiliations:** 1 Centre for Integrated Materials, Processes & Structures, Department of Mechanical Engineering, University of Bath, Claverton Down, Bath BA27AY, UK; 2 Centre for Advanced Structural Ceramics, Imperial College London, London SW72AZ, UK; 3 EMD Ltd. The Old Manse, 29 St Mary St, Ilkeston, Derbyshire DE78AB, UK; 4 Centre for Digital, Manufacturing & Design (dMaDe), Department of Mechanical Engineering, University of Bath, Claverton Down, Bath BA27AY, UK; 5 Department of Electronic and Electrical Engineering, University of Bath, Claverton Down, Bath BA27AY, UK

**Keywords:** applied sciences, energy storage, materials science

## Abstract

Piezoelectric energy harvesting of water flow offers a promising approach for directly powering Internet-of-Things (IoT) sensors used in water environment monitoring. However, its voltage and power outputs remain limited under practical flow conditions, especially in turbulent and low-velocity flows. To address this challenge, this work presents the first demonstration of a water flow energy harvester integrated with *d*_33_-mode and porosity-engineered lead-free Ba_0.85_Ca_0.15_Zr_0.1_Ti_0.9_O_3_ (BCZT) piezoelectric ceramics. The performance of the harvester was experimentally evaluated in turbulent water flow at a low velocity of 0.1 m/s. During a capacitor charging test, the porous BCZT harvester achieved a 2.3-times increase in the saturated charging voltage (*V*_*charge*_) and a 5.1-fold increase in the charging power compared to the dense BCZT harvester. This work fills the knowledge gap related to piezoelectric harvesters in low-velocity turbulent flow, demonstrating the critical role of material design in water flow energy harvesting, and promoting the development of self-powered water monitoring technologies.

## Highlights

• Porous lead-free BCZT piezoelectric ceramics are applied to water-flow harvesting
• *d*_33_-mode was integrated into a cantilever beam based energy harvester
• Porous BCZT yields 2.3× voltage and 5.1× power output compared to dense material
• Our work fills the knowledge gap in harvesters for low-velocity turbulent water flow

## Graphical abstract

**Figure:**
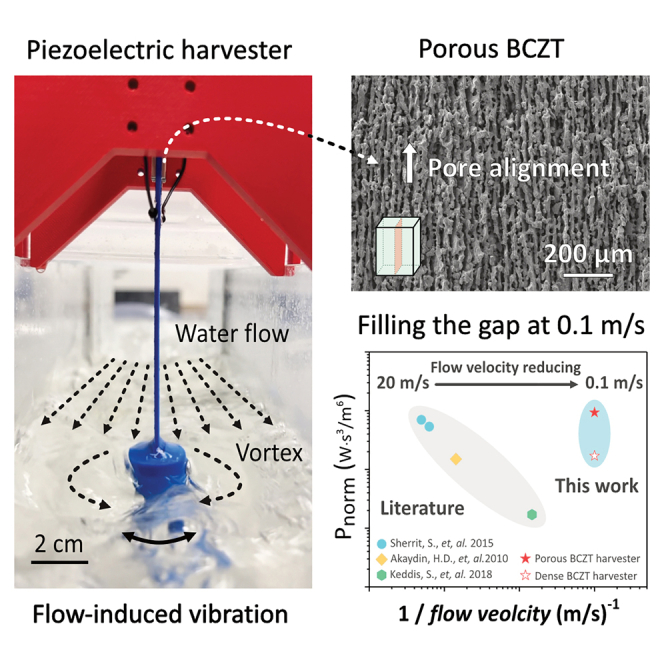

## Introduction

Recently, the monitoring of our water environment has received increasing attention due to its essential role in tackling emerging challenges such as water resource management,^1^ water pollution,^2^ climate change,^3^ and aquatic ecosystem protection.^4^ The rapid development and implementation of the Internet of Things (IoT) and smart sensor technologies^5^ offers a new approach to water environment monitoring, enabling remote operation,^6^ large-scale deployment,^7^ and real-time data collection.^8^ Nevertheless, powering IoT sensors in water environments remains a major challenge, in particular when operating in remote areas beyond the reach of the power grid. In this case, water sensors typically rely on replaceable batteries, leading to high costs associated with battery replacement, maintenance and their final disposal.^7^

Piezoelectric energy harvesting is an emerging concept that utilizes piezoelectric materials as a transducer to convert mechanical energy from the environment into useful electrical energy.^9^ This technique offers a promising solution for self-powered IoT sensors in water environments, enabling them to convert renewable ambient mechanical energy from water flow into electrical energy as a reliable power supply. Due to their advantages of a high voltage output at low flow velocities, a high energy density for operation with small material volumes, and a long service life,^10^ researchers have studied piezoelectric harvesters with a particular focus on the flow-induced vibration (FIV) mechanisms,^11^ harvester geometry configurations,^12^ and the integration of magnets to tune the resonance frequency and oscillation amplitude generated by fluid-induced forces.^13^

Despite recent advances in optimized FIV mechanisms, laminar flow conditions have typically been assumed in research studies to date,^10^^,^^11^^,^^12^^,^^13^ where water flows in parallel layers with a uniform direction and velocity without any turbulence. Idealized laminar water flow allows the most efficient operation of FIV mechanisms, ensuring a high harvester output and reliable test accuracy. However, in practical water environments, such as rivers, turbulent water flow is far more common.^14^ The presence of turbulence introduces irregular flow velocities and multiple flow directions, which significantly reduces the efficiency of the FIV, consequently limiting both the mechanical energy input that is supplied to the piezoelectric harvester and subsequently the generated voltage and power output of the system.^15^^,^^16^ Furthermore, since the energy density (*E*) of water flow is proportional to the cube of the flow velocity (*v*), i.e., *E* ∝ *v*^3^, a harvester’s output typically becomes too low to measure when the flow velocity is reduced to 0.1–0.3 m/s.^17^^,^^18^^,^^19^ This velocity range is common for rivers in flat regions such as plains.^20^^,^^21^ For example, a flow velocity of 0.27 m/s has been used in fisheries and aquaculture estimation by the Food and Agriculture Organization (FAO) of the United Nations.^22^ The poor performance of piezoelectric harvesters in turbulent, low-velocity conditions leads to two major issues. First, a low voltage output may fail to overcome the voltage drop during rectification of any alternating signal produced by a piezoelectric, which can be as high as 1.4 V,^23^^,^^24^ thereby preventing an effective DC output. Second, the insufficient power output generated by the harvester may be inadequate to sustain sensor operation, rendering the water monitoring system ineffective or leading to less frequent data collection. Consequently, research on piezoelectric harvesters in turbulent flows is limited and primarily focuses on a relatively high flow velocity range from 0.75 to 20 m/s.^25^^,^^26^ This creates a research gap regarding piezoelectric harvesters functioning in low-velocity turbulent flows.

As pointed out in ref.^10^, a fundamental factor for the low voltage and power output is the slow development of the piezoelectric materials used in these harvesters. The piezoelectric material used in a harvester significantly impacts two processes during energy harvesting^27^: (1) the energy *extraction* process, which determines the amount of mechanical energy that is input into the piezoelectric, which the harvester can capture; and (2) the energy *conversion* process, which governs the efficiency of converting mechanical energy into electrical energy. The piezoelectric energy harvesting figure-of-merit (*FoM*_*ij*_) serves as a key parameter for evaluating the overall performance of a piezoelectric material in these two processes. For water flow piezoelectric energy harvesters, there has been no significant improvement in the *FoM*_*ij*_ of commonly used piezoelectric materials since the year 2000.^10^ Moreover, lead-based piezoelectric materials have been predominantly used in approximately 70% of water flow piezoelectric harvesters,^10^ such as lead zirconate titanate (PZT) and macro fiber composites (MFCs) that employ PZT fibers. This widespread reliance on lead-based materials presents another challenge for the development of water flow piezoelectric harvesters. Since lead is harmful to the environment, governments worldwide are also increasingly imposing restrictions on its use in electronic product manufacturing.^28^

**Figure 1:**
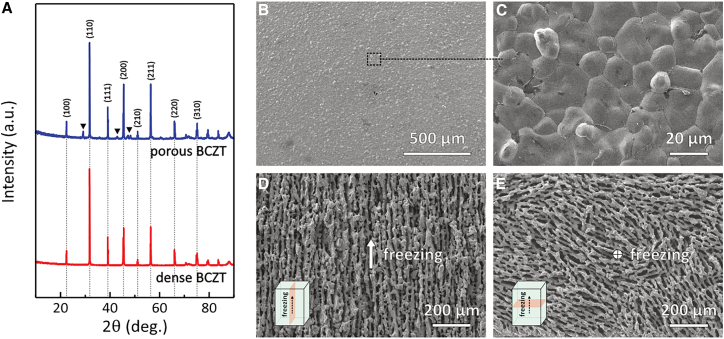
Characterization of BCZT ceramics (A) XRD patterns and microscopies of the BCZT ceramics: (B) dense BCZT ceramics and (C) its local magnification; freeze-casting porous BCZT ceramics’ (D) transverse- and (E) cross-sections. Direction of freezing is indicated. The lengths of the scale bars in Figures (B), (C), (D), and (E) are 500, 20, 200, and 200 μm, respectively.

**Figure 2:**
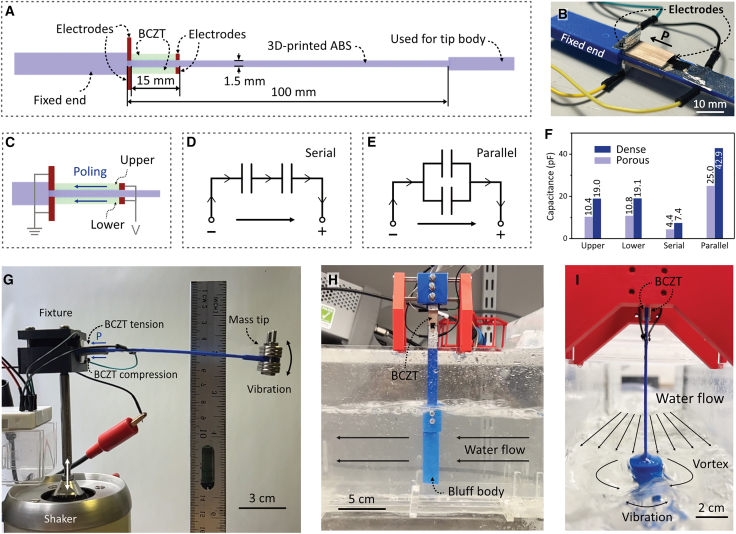
Schematic and experimental setup of the BCZT-based piezoelectric energy harvester (A–F) (A) Schematic of piezoelectric energy harvester structure, (B) enlarged image of the BCZT sheets installed on the cantilever beam, (C) BCZT poling direction, electrical connection configurations of serial (D) and parallel (E) modes and their capacitance (F). (G–I) (G) Experimental setup for energy harvesting evaluation using a shaker as the energy input. Water-flow induced vibration energy harvesting test setup from (H) a parallel view and (I) a perpendicular view relative to the water flow direction.

It has been shown that the combination of lead-free piezoelectric ceramics Ba_0.85_Ca_0.15_Zr_0.1_Ti_0.9_O_3_ (BCZT) and porosity engineering was demonstrated to be an effective approach to addressing both the low *FoM*_*ij*_ of conventionally processed piezoelectric materials and the reliance on lead-based piezoelectric materials.^29^ In this study, BCZT ceramics are fabricated (Figure 1 and Table 1) and subsequently integrated into a cantilever beam to function as a piezoelectric energy harvester (Figure 2). The harvester is experimentally evaluated in a water channel with turbulent flow at a low flow velocity of approximately 0.1 m/s (Videos S1 and S2). Compared to a harvester based on a dense BCZT ceramic, the porous BCZT harvester increased the saturated charging voltage of the capacitor (*V*_*oc*_) by 250% and increased the charging power by 410%, enabling a reliable DC power supply for electronic devices requiring an input voltage below 2.1 V, successfully filling the gap of the piezoelectric harvester in low-velocity turbulent flow. By taking advantage of lead-free porous piezoelectric materials, two critical challenges in water flow piezoelectric energy harvesting are successfully addressed, namely, the limited harvesting figure of merit, *FoM*_*ij*_, and the reliance on lead-based materials. This work therefore offers a novel strategy for improving the performance of water flow piezoelectric harvesters in turbulent, low-velocity flow conditions, thereby promoting the development of self-powered water monitoring systems based on piezoelectric energy harvesting.

**Table 1:** Properties of the poled BCZT sheets after aging for 72 h (*d*_33_ = longitudinal piezoelectric charge coefficient, piezoelectric voltage constant $g_{33}=d_{33}/\varepsilon_{33}^{T}$ where $\varepsilon_{33}^{T}/\varepsilon_{0}$ = relative permittivity at constant stress, piezoelectric energy harvesting figure-of-merit *FoM*_33_ = *d*_33_ × *g*_33_, $k_{33}^{2}$ = electromechanical coupling coefficient, *Y* = Young’s modulus, *Q* = mechanical quality factor)

Samples	Mass (g)	Open porosity (vol. %)	Capacitance (pF)	*tanδ* (%)	*d*_33_ (pC/N)	*g*_33_ (10^−3^ V·m/N)	*FoM*_33_ (10^−12^ m^2^/N)	$k_{33}^{2}$ (%)	*Y* (GPa)	*Q*
Dense BCZT	1.16	1.9	19.1	1.80	305	15.6	4.76	19.2	40.4	151
Porous BCZT	0.62	50.3	10.6	1.33	412	40.2	16.6	38.2	23.0	117

Video S1. Flow-induced vibration of the harvester in a water flow channel

Video S2. Electrical output measurement of the water flow energy harvesting test

## Results

### Free-damped oscillation

**Figure 3:**
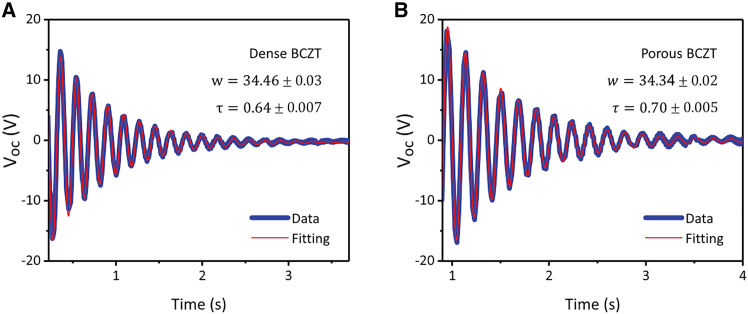
Open-circuit voltage (*V*_*oc*_) output under damped-free vibration conditions (A) Using dense BCZT and (B) using porous BCZT ceramics. Data are represented as mean ± standard deviation.

To characterize the mechanical and damping properties of the piezoelectric energy harvester, a free-damped vibration test was initially conducted. During testing, a static displacement preload was applied to the mass tip to induce bending of the cantilever beam. Upon removing the preload, the mass tip was released, allowing the cantilever beam to freely oscillate without external excitation or restriction. Figure 3 shows the open-circuit voltage output (*V*_*oc*_) of the harvester during free-damped oscillation. Since the voltage output of a piezoelectric material is proportional to its mechanical strain, the *V*_*oc*_ in Figure 3 semi-quantitatively represents the oscillation deflection (*x*) of the cantilever beam, where:

(Equation 1) $$\begin{array}{c} x\;\propto \;V_{oc} \end{array}$$

For free-damped oscillation, the deflection (*x*) as a function of time (*t*) can be expressed as

(Equation 2) $$\begin{array}{c} x={Ae}^{-\frac{t}{\tau }}\;\cos\left(wt+\delta \right)+C \end{array}$$

where *A* is the oscillation amplitude, *τ* is the damping ratio, *w* is the oscillation angular speed, *δ* is the phase different between the *x* and the force acting on the cantilever beam (including the force due to the damping effect and the bending stiffness), and *C* accounts for the steady-state offset of *x* which was not zero due to presence of the mass at the tip.

By fitting the time-dependency of the *V*_*oc*_ data in Figure 3 to Equations 1 and 2, the parameters *w* and *τ* can be determined. These parameters were subsequently used to calculate the damping torque coefficient (*r*) and bending torque stiffness (*c*) of the harvester, as follows:

(Equation 3) $$\begin{array}{c} r=2I_{m}/\tau \end{array}$$

(Equation 4) $$\begin{array}{c} c=m\left[w^{2}+{\left(r/2I_{m}\right)}^{2}\right] \end{array}$$

where *I*_*m*_ is the inertial moment of mass

(Equation 5) $$\begin{array}{c} I_{m}=\int_{0}^{L_{0}}L^{2}dm \end{array}$$

Here, *dm* is the differential mass element of the cantilever beam, *L* is the distance from *dm* to the fixed end, and *L*_0_ is the total length of the cantilever beam. Since *I*_*m*_ can be influenced by the *m* of the harvester, the difference in mass of the dense BCZT (1.16 g per sheet) and porous BCZT (0.62 g per sheet) must be considered. Nevertheless, given that the attached mass tip weighed 27 g—over 20 times heavier—and was fixed at the free tip with an average distance *L* of 120 mm, which was approximately 15 times longer than the average *L* of the mounted BCZT sheets, the influence of the mass difference between dense and porous BCZT sheets became negligible. Specifically, the resulting difference in *I*_*m*_ is estimated to be as low as 0.3%.

The calculated values of *r* and *c* are presented in Table 2. The results indicated that the cantilever beam harvester incorporating porous BCZT exhibited bending stiffness comparable to that of the harvester with dense BCZT, despite the Young’s modulus of the individual porous BCZT sheet being approximately 50% lower than that of its dense counterpart. This is attributed to the fact that the overall bending stiffness of the cantilever beam harvester was primarily governed by the 3D-printed ABS substrate (with a Young’s modulus of ∼2 GPa), as shown in Figure 2A, rather than the BCZT sheets themselves due to their significantly higher Young’s modulus ranging from 23 to 40 GPa. The resonance frequency (*f*_0_) of the harvester can be calculated using

(Equation 6) $$\begin{array}{c} f_{0}=\sqrt{c/I_{m}} \end{array}$$

**Table 2:** Mechanical properties of the harvesters at resonance with dense and porous (50 vol. % porosity) BCZT sheets (*r* = damping torque coefficient and *c* = bending torque stiffness)

Properties	Dense BCZT	Porous BCZT
*r* (N·m·(rad/s)-1)	2.18 ± 0.06 × 10^−3^	1.99 ± 0.03 × 10^−3^
*c* (N·m·(rad)-1)	0.833 ± 0.03	0.827 ± 0.02
Bending stiffness (N/mm)	0.0687 ± 0.03	0.0682 ± 0.02
Mechanical resonance frequency of the first bending mode (Hz)	5.49 ± 0.005	5.47 ± 0.003

Data are represented as mean ± standard deviation.

The values of *f*_0_ were found to be 5.49 Hz and 5.47 Hz for the harvesters based on the dense and porous BCZT ceramics, respectively. These values guided the study on the frequency dependency of the harvester during the shaker tests.

### Shaker tests (dry testing)

**Figure 4:**
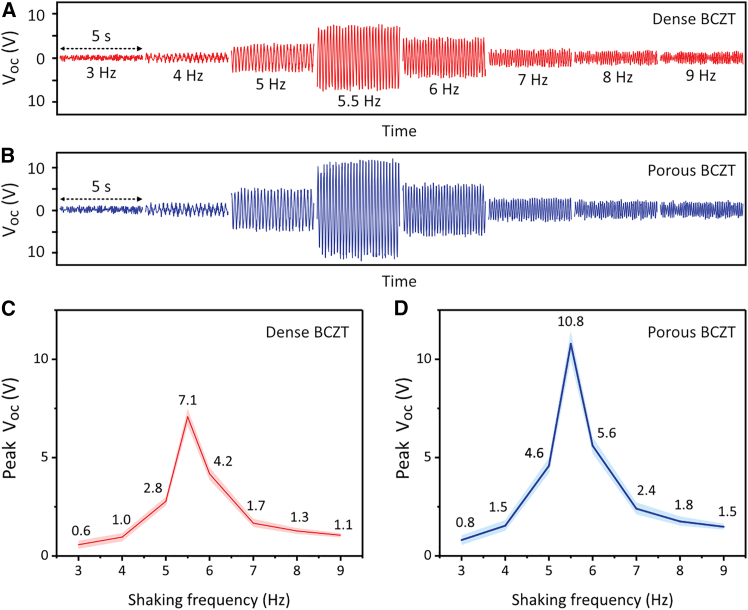
Frequency dependency of open-circuit voltage (*V*_*oc*_) output (A and C) Using dense BCZT and (B and D) using porous BCZT ceramics, respectively, during the dry shaker test across a range of frequencies. BCZT sheets were connected in parallel. Data are represented as mean ± standard deviation.

The first objective of the shaking test was to determine the frequency dependency of the harvester, in which the two BCZT sheets of the harvester were electrically connected in parallel. This was used to understand their sensitivities to various frequencies prior to being tested in a turbulent water flow. The shaker was driven by a sinusoidal voltage waveform with a constant peak-to-peak value and a controllable frequency. As shown in Figures 4A and 4B, the shaking frequency significantly influenced the open-circuit voltage (*V*_*oc*_) that was generated. By fitting the *V*_*oc*_ data to a sinusoidal function of time, the peak values of the *V*_*oc*_ were determined, as shown in Figures 4C and 4D. The results showed that the peak *V*_*oc*_ increased with shaking frequency, reaching a maximum at approximately 5.5 Hz, and then decreased as the shaking frequency further increased. This indicated that the harvester exhibited a resonance frequency at around 5.5 Hz, where the maximum oscillation amplitude was achieved. This finding is consistent with the resonance frequency calculated in Table 2. Based on these results, the following studies, which investigated the impact of different electrical connection configurations of the BCZT sheets, were conducted at the resonance frequency of 5.5 Hz to maximize the output and ensure accuracy of testing.

**Figure 5:**
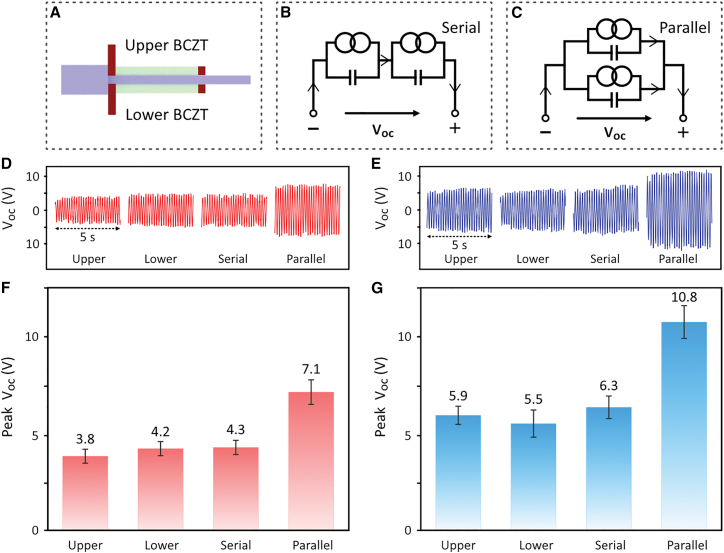
Open-circuit voltage (*V*_*oc*_) output under different electrical connection configurations (A) Schematic of upper and lower BCZT of piezoelectric energy harvester, and their electrical connection configurations of serial (B) and parallel (C) modes. Open-circuit voltage (***V***_***oc***_) output and corresponding peak ***V***_***oc***_ values of the piezoelectric energy harvester at a shaking frequency of 5.5 Hz, using (D and F) dense BCZT and (E and G) porous BCZT ceramics, respectively, with configurations of individual upper and lower BCZT sheets, and serial and parallel connections between them. Data are represented as mean ± standard deviation.

At the optimized shaking frequency of 5.5 Hz, the upper and lower BCZT sheets, see Figure 5A, were electrically connected in serial and parallel modes, see Figures 5B and 5C in which an individual piezoelectric material is represented by an AC current source in parallel with its internal electrical capacitance. The impact of the electrical connection configurations on the harvester’s output was investigated. Figures 5D and 5E show the measured open-circuit voltage (*V*_*oc*_) for the individual upper and lower BCZT sheets, as well as their serial and parallel connections. By fitting the data to a sinusoidal waveform function, the peak *V*_*oc*_ values were obtained, as shown in Figures 5F and 5G. The individual upper and lower BCZT sheets exhibited similar peak *V*_*oc*_ values. Notably, the peak *V*_*oc*_ remained nearly unchanged in the serial connection compared to that of the individual BCZT sheets, but the peak *V*_*oc*_ doubled in a parallel connection. This result appears inconsistent with conventional principles of capacitor behavior: in a series configuration with a constant amount of generated charge, the total capacitance is halved and the voltage is doubled, whereas in a parallel configuration, the total capacitance is doubled and the voltage is halved. The mechanism underlying this discrepancy remains unclear in the present study, and further investigation is expected. The use of porous BCZT resulted in an approximately 50% increase in the *V*_*oc*_ compared to the dense BCZT counterpart for all four connection configurations.

**Figure 6:**
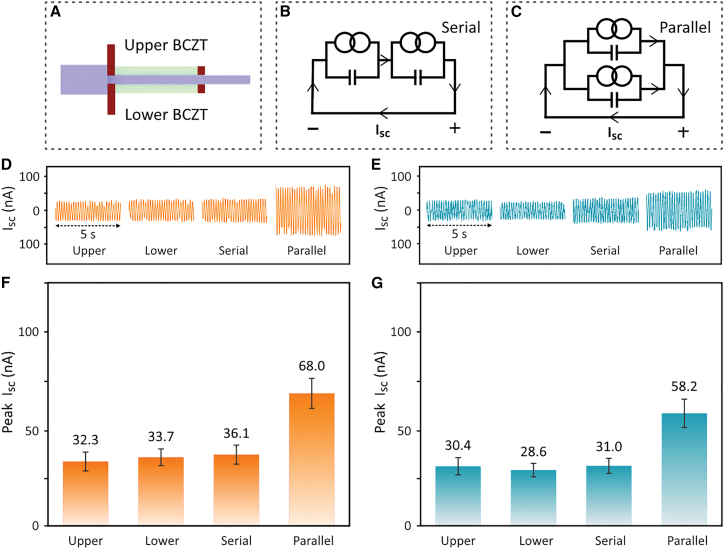
Short-circuit current (*I*_*sc*_) output under different electrical connection configurations (A) Schematic of upper and lower BCZT of piezoelectric energy harvester, and their electrical connection configurations of serial (B) and parallel (C) modes. Short-circuit current (***I***_***sc***_) output and corresponding peak ***I***_***sc***_ values of the piezoelectric energy harvester at a shaking frequency of 5.5 Hz, using (D and F) dense BCZT and (E and G) porous BCZT ceramics, respectively, with configurations of individual upper and lower BCZT sheets, and serial and parallel connections between them. Data are represented as mean ± standard deviation.

Similarly, the effect of the electrical connection configuration on the short-circuit current (*I*_*sc*_) output was examined, with electrically connecting the upper and lower BCZT sheets, see Figure 6A, in serial and parallel modes, see Figures 6B and 6C. The *I*_*sc*_ result in time domain is shown in Figures 6D and 6E. The corresponding peak *I*_*sc*_ values are summarized in Figures 6D and 6F. The individual upper and lower BCZT sheets generated comparable peak *I*_*sc*_ values. Interestingly, the serial connection yielded a peak *I*_*sc*_ value that was comparable to that of the individual sheets, while the parallel connection approximately doubled the peak *I*_*sc*_ output. In addition, the use of porous BCZT led to a 14% lower *I*_*sc*_ compared to the dense BCZT. Based on these findings, the subsequent shaking tests for energy harvesting were conducted at the resonance frequency of 5.5 Hz, with the BCZT sheets connected in parallel to maximize the output and ensure the accuracy of the results.

To examine the maximum power output (*P*_*out*_), the harvester was connected to a purely resistive circuit. The *P*_*out*_ was calculated based on the voltage output (*V*_*out*_) and the load resistance (*R*_*load*_):

(Equation 7) $$\begin{array}{c} P_{out}={V_{out}}^{2}/R_{load} \end{array}$$

**Figure 7:**
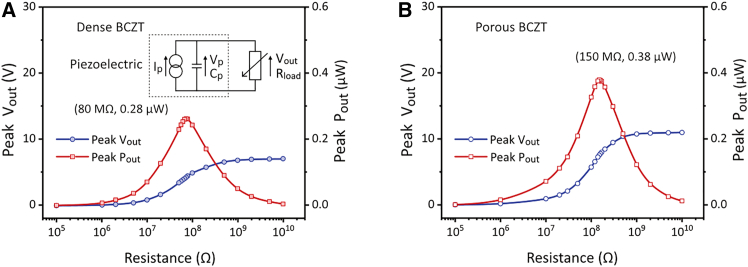
Voltage output (*V*_*out*_) and power output (*P*_*out*_) in purely resistive circuits (A) Using dense BCZT and (B) using porous BCZT ceramics, with parallel-connected BCZT sheets and at a shaking frequency of 5.5 Hz.

In Figure 7, the piezoelectric material is represented by an AC current source (*I*_*p*_) in parallel with its internal electrical capacitance (*C*_*p*_), and an AC voltage *V*_*p*_ across them. With an increase in *R*_*load*_, *V*_*out*_ continuously increased to the value of the open-circuit voltage output. The maximum *P*_*out*_ of the harvesters with dense and porous BCZT ceramics were found at the *R*_*load*_ of 80 MΩ and 150 MΩ, respectively. The harvester with porous BCZT generated a peak *P*_*out*_ that was 1.4 times higher than that of the dense BCZT harvester. For purely resistive circuits, the optimized power output is typically assumed to be achieved when the load resistance *R*_*load*_ matches the internal electrical impedance *Z*_*piezo*_ of the piezoelectric harvester^10^:

(Equation 8) $$\begin{array}{c} Z_{piezo}=1/2\pi fC_{p} \end{array}$$

where *f* is the vibration frequency, and *C*_*p*_ is the capacitance of the harvester. Notably, the experimentally measured optimized *R*_*load*_ for the dense and porous BCZT harvesters was significantly lower than the predicted optimum *R*_*load*_ calculated through Equation 8 of 675 MΩ and 1160 MΩ, respectively. Further work is needed to determine the cause of the discrepancy between the optimized *R*_*load*_ values obtained by the two methods.

**Figure 8:**
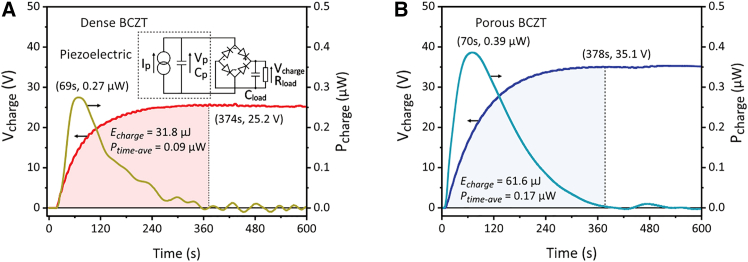
Voltage across a charged capacitor (*V*_*charge*_) and charging power (*P*_*charge*_) in energy harvesting circuits (A) Using dense BCZT and (B) using porous BCZT ceramics, with parallel-connected BCZT sheets and at a shaking frequency of 5.5 Hz.

In practical energy harvesting applications, the alternating voltage (AC) output cannot directly serve as the power supply for electronic devices, such as sensors and data transmission modules, due to its AC output. Therefore, the performance of the harvester was further evaluated using a standard energy harvesting circuit as shown in Figure 8 in which the AC output was rectified by a full-bridge rectifier composed of four 1N4007 diodes with a voltage drop of approximately 0.6–0.7 V per diode. This setup enabled a loading capacitor (*C*_*load*_, 100 nF) to be continuously charged, serving as a DC power supply for external electronic devices. The loading resistance (*R*_*load*_) was equal to the internal electrical impedance of the electrometer, which exceeded 1 TΩ. The voltage (*V*_*charge*_) across the *C*_*load*_ over time, along with the real-time charging power (*P*_*charge*_) calculated as

(Equation 9) $$\begin{array}{c} P_{charge}=\frac{d}{dt}\left(\frac{{C_{load}V_{charge}}^{2}}{2}\right) \end{array}$$

The maximum *P*_*charge*_ of the harvester with dense and porous BCZT were observed at 69 s (0.27 μW) and 70 s (0.39 μW), respectively. Based on the electrical energy stored in the capacitor:

(Equation 10) $$\begin{array}{c} E_{charge}=\frac{1}{2}C_{load}V_{charge}^{2} \end{array}$$

the time-average charging power is calculated as

(Equation 11) $$\begin{array}{c} P_{time-ave}=E_{charge}/T \end{array}$$

where *T* represents the time at which *P*_*charge*_ reduced to zero, namely the moment at which *V*_*charge*_ reached the saturated value. This was 372 s and 378 s for the harvesters with dense and porous BCZT, respectively, as shown in Figures 8A and 8B. Compared to the harvester based on the dense BCZT, the porous BCZT harvester exhibited a 39% higher saturated *V*_*charge*_, 44% higher maximum *P*_*charge*_, and 89% higher *P*_*time*-*ave*_, demonstrating the considerable enhancements in the energy harvesting performance of the porous material.

As shown in Table 1, the introduction with porosity of low permittivity resulted in the porous BCZT exhibiting a piezoelectric voltage constant *g*_33_, a measure of the electric field produced per unit stress, which was 2.6-times higher than the dense materials. The porous BCZT also exhibited a piezoelectric energy harvesting figure-of-merit *FoM*_33_ value, a measure of the energy density of the material for an applied stress that was 3.5-times higher than the dense BCZT. Notably, compared to the increase in the *g*_33_ and *FoM*_33_, the enhancements in the voltage and power output were smaller in the shaking test of 1.5- and 1.4-times, respectively; see Figures 5 and 7. This difference could be attributed to the fact that during the shaking test the external mechanical excitation was applied by the shaker to the fixed end of the cantilever, rather than the mass tip, as seen in Figure 2E. This condition was closer to a strain-driven regime,^30^ rather than a stress-driven regime, where the mechanical strain input of the piezoelectric material remained constant. Consequently, the lower the stiffness of the porous BCZT resulted in a reduction in the bending stress applied to the material. This was also reflected in the short-circuit current (*I*_*sc*_) results, as shown in Figure 6. The *I*_*sc*_ is the changing rate of the piezoelectric charge as

(Equation 12) $$\begin{array}{c} I_{sc}=\frac{dQ}{dt}=\frac{d\left(F\left(t\right)d_{33}\right)}{dt} \end{array}$$

where *F*(*t*) is the force subjecting to the piezoelectric material as a function of time. For a sinusoidal form of *F*(*t*), it can be expressed as

(Equation 13) $$\begin{array}{c} F\left(t\right)=\mathrm{Asin}\left(2\pi ft\right) \end{array}$$

where *A* and *f* are the force amplitude and frequency, respectively. By fitting Equation 13 into Equation 12, *I*_*sc*_ becomes

(Equation 14) $$\begin{array}{c} I_{sc}=2\pi fAd_{33}\;\cos\left(2\pi ft\right) \end{array}$$

Therefore, *I*_*sc*_ is proportional to the product of the force amplitude (*A*) and frequency (*f*), and piezoelectric charge coefficient (*d*_33_). This indicates that the bending stress applied to the cantilever beam with porous BCZT was only 63% of that applied to the dense counterpart. Consequently, the observed variation amplitudes of the voltage and power output were smaller than those of the *g*_33_ and *FoM*_33_.

### Water flow energy harvesting test (wet test)

**Figure 9:**
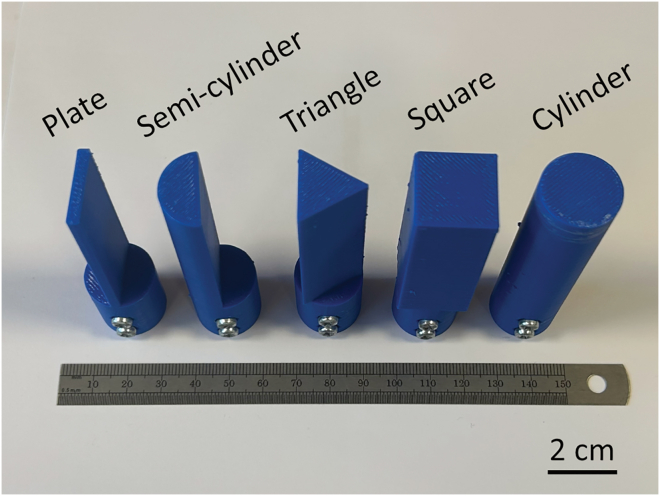
Bluff bodies connected to the free end of the harvester, featuring five different cross-sectional geometries: plate, semi-cylinder, triangle, square, and cylinder

**Figure 10:**
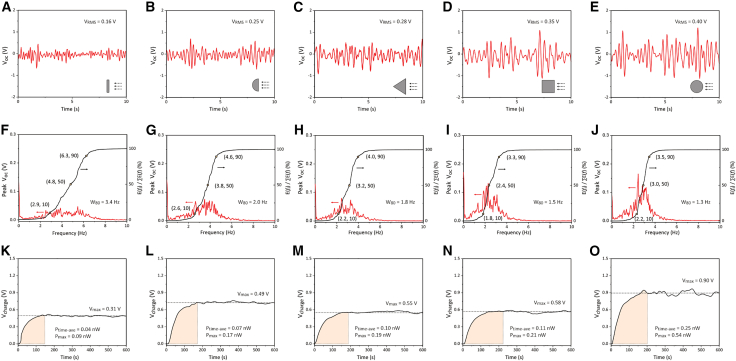
Output of the piezoelectric energy harvester in a water flow using dense BCZT ceramics With five bluff body configurations attached to the tip of the cantilever beam, including plate, semi-cylinder, triangle, square and cylinder. (A–E) Open-circuit voltage output (***V***_***oc***_), (F–J) Fast-Fourier transformation (FFT) of the ***V***_***oc***_ and the distribution of the energy contribution across the oscillation frequencies from 0 to 10 Hz, and (K–O) charging behavior of a capacitor with connection to the harvester and a full-wave rectifier energy harvesting circuit.

**Figure 11:**
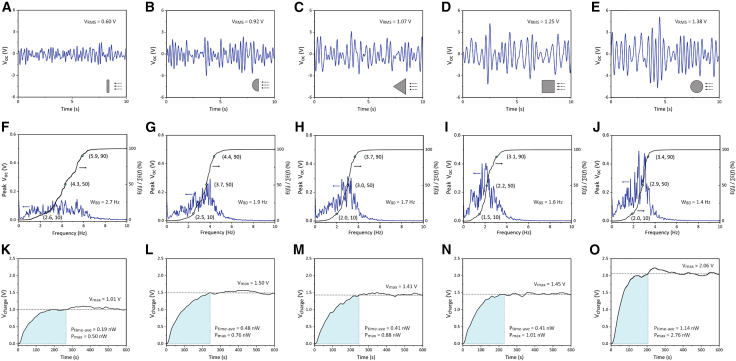
Output of the piezoelectric energy harvester in water flow using porous BCZT ceramics With five bluff body configurations attached to the tip of the cantilever beam, including plate, semi-cylinder, triangle, square and cylinder. (A–E) Open-circuit voltage output (***V***_***oc***_), (F–J) Fast-Fourier Transformation (FFT) of the ***V***_***oc***_and the distribution of the energy contribution across the oscillation frequencies from 0 to 10 Hz, and (K–O) charging behavior of a capacitor with connection to the harvester and a full-wave rectifier energy harvesting circuit.

The two harvesters were then tested in a water flow channel to assess their performance in practical water environments. During the water flow energy harvesting test, the mass tip connected to the cantilever beam was replaced by a bluff body, enabling the cantilever beam to be mechanically excited by the water flow through flow-induced vibrations (FIVs). Five bluff body geometries with the same effective width of 20 mm perpendicular to the flow direction were designed to optimize the FIV performance, including plate, semi-cylinder, triangle, square and cylinder, as shown in Figure 9. The open-circuit voltage (*V*_*oc*_) of the harvester with dense and porous BCZT was measured, as shown in Figures 10A–10E and Figures 11A–11E, respectively, which provided the time-domain information about the vibration amplitude. The *V*_*oc*_ data were processed using fast Fourier transformation (FFT) to determine the vibration frequency, as presented in Figures 10F–10J and 11F–11J. During the capacitor charging test, the harvesters were connected to a standard energy harvesting circuit to charge a 100 nF capacitor. The charging voltage (*V*_*charge*_) across the capacitor are shown in Figures 10K–10O, and the calculated time-average charging power (*P*_*time*-*ave*_) and maximum charging power (*P*_*max*_) are illustrated and Figures 11K–11O.

As shown in the time-domain *V*_*oc*_ data in Figures 10A–10E and 11A–11E, the FIV of all the harvesters tested exhibited non-periodic patterns. To quantitatively evaluate the *V*_*oc*_ level, the root-mean-square (RMS) value was used:

(Equation 15) $$\begin{array}{c} V_{RMS}=\frac{\sqrt{\sum_{n}{V_{oc,i}}^{2}}}{n} \end{array}$$

where *n* is the number of the data points collected over a period of 60 s with a scanning frequency of 50 Hz, and *V*_*oc*,*i*_ is the value of the *V*_*oc*_ at the point *i*. The cross-section configuration of the bluff body significantly influenced the *V*_*RMS*_. Specifically, the plate bluff body resulted in the lowest *V*_*RMS*_, indicating the smallest FIV amplitude. In comparison, the semi-cylinder and triangle bluff bodies increased the *V*_*RMS*_ by approximately 60 and 80%, respectively. The square and cylinder bluff bodies further enhanced the *V*_*RMS*_ by approximately 110% and 140%, respectively, compared to the *V*_*RMS*_ with the plate bluff body. The enhancement on the voltage output should be due to the increased vibration amplitude, which could result from stronger fluid-solid interactions of the square and cylinder bluff bodies, as well as their greater masses, compared to the other bluff bodies. In addition, compared to the harvester with dense BCZT, the use of porous BCZT resulted in a 245%–282% improvement in *V*_*RMS*_.

The FFT analysis of the *V*_*oc*_ data provided insight into the frequency modes of the flow-induced vibration (FIV) behavior. Since no dominant peak was observed in any of the FFT results, no harvester tested was excited by a specific FIV mechanism, such as vortex-induced vibration (VIV), galloping or wake flow-induced vibration (WIV). Instead, the observed vibrations were primarily attributed to turbulence-induced vibration (TIV).^10^ The energy harvested at each specific frequency mode (*f*) was semi-quantitively expressed as

(Equation 16) $$\begin{array}{c} E\left(f\right)={V_{oc}\left(f\right)}^{2}f \end{array}$$

The contribution of the vibration at a specific *f* mode to the total energy harvesting performance was determined as *E*(*f*) / ∑*E*(*f*). As *f* increased from 0 Hz to 10 Hz, the ratio of the accumulated energy contribution *E*(*f*) / ∑*E*(*f*) increased from 0% to 100%, indicating that all the FIV frequencies fell within this range. The frequency at which *E*(*f*) / ∑*E*(*f*) reached 50% was marked in Figures 10F–10J and Figures 11F–11J to indicate the primary frequency mode of the FIV. The frequency distribution of the FIV was evaluated using the index *W*_80_, defined as the bandwidth between the frequencies at which *E*(*f*) / ∑*E*(*f*) equaled 10% and 90%. Among the different bluff body configurations, the square bluff body resulted in the lowest vibration frequency mode (∼2.3 Hz). The cylinder bluff body enabled the smallest *W*_80_ (∼1.6 Hz). The plate bluff body exhibited the highest vibration frequency mode (∼4.5 Hz) and the largest *W*_80_ (∼3 Hz). Moderate frequency modes (3.0–3.8 Hz) and *W*_80_ (1.7–2.0 Hz) were observed in the harvester with the semi-cylinder and triangle bluff bodies. In addition, the use of porous BCZT slightly reduced the vibration frequency mode of 0.1–0.5 Hz, compared to the dense BCZT counterpart.

During the capacitor charging test, all the capacitors were fully charged in 150–250 s, reaching the maximum voltage (*V*_*charge*_) and remaining stable over time. The plate bluff body resulted in the lowest saturated *V*_*charge*_ and the lowest charging power, including both time-average value (*P*_*time*-*ave*_) and maximum value (*P*_*max*_). In contrast, the harvester with the cylinder bluff body showed the highest saturated *V*_*charge*_ and charging power. Notably, the harvester with the porous BCZT ceramic demonstrated substantial improvement in energy harvesting performance compared to the harvester with dense BCZT using the corresponding bluff body configuration, including a 130%–230% increase in the saturated *V*_*charge*_ and 350%–450% enhancement in the charging power, which highlights the significant advantage of porous BCZT in practical water flow energy harvesting.

## Discussion

### Benefits of using porous BCZT

The benefits of using porous BCZT in water flow piezoelectric harvesting are now summarized with particular focus on (1) the impact of the increased voltage output on the AC rectification, (2) the impact of the decreased capacitance on the charging power output, and (3) the impact of the decreased stiffness and damping coefficient on the flow-induced vibration.

(i) Advantages of using porous BCZT in AC rectification by increasing *g*_33_ and *V*_*oc*_

For practical applications of water flow piezoelectric energy harvesting, AC rectification is essential to obtain the required DC power supply. During the sinusoidal-waveform shaker test (dry test), the saturated voltage across the charged capacitor (*V*_*charge*_) was analyzed based on the result from the standard energy harvesting circuit (Figure 8). According to ref.^31^^,^^32^, the saturated value of *V*_*charge*_ can be analytically expressed as

(Equation 15) $$\begin{array}{c} V_{charge}=\frac{2}{\pi }\times \frac{I_{out}R_{load}}{1+4fC_{piezo}R_{load}} \end{array}$$

where *f* is the frequency, *I*_*out*_ is the current output of the harvester, *R*_*load*_ is the load resistance, and *C*_*piezo*_ is the capacitance of the piezoelectric material. Therefore, to enhance *V*_*charge*_, a high ratio of *I*_*out*_ / *C*_*piezo*_ is essential. Notably, this ratio is positively correlated with the piezoelectric voltage constant $g_{33}=d_{33}/\varepsilon_{33}^{T}$, which demonstrates the advantage of the high *g*_33_ value of the porous BCZT (see Table 1) in improving its experimentally measured *V*_*charge*_. Furthermore, by considering the open-circuit voltage (*V*_*oc*_) analyzed based on the result from the purely resistive circuit (Figure 7), a quantitative relationship between *V*_*oc*_ and *V*_*charge*_ is approximated as

(Equation 16) $$\begin{array}{c} V_{charge}=4\left({\text{Peak}\;V}_{oc}-V_{drop}\right) \end{array}$$

where *V*_*drop*_ represents the voltage drop caused by each diode in the rectifier. Since the value of *V*_*drop*_ remains constant, using porous BCZT tends to provide a greater improvement in *V*_*charge*_ when the difference Peak*V*_*oc*_-*V*_*drop*_ for the dense material is close to zero, i.e., with a relatively small *V*_*oc*_ of the dense counterpart, which typically occurs when the mechanical input of the harvester is weak. As shown in Figures 10A and 11A, the harvester with a plate bluff body had relatively weak mechanical input, resulting in a low *V*_*oc*_ with root-mean-square values (RMS) of 0.2–0.6 V. Consequently, using a porous BCZT led to a considerable enhancement of 226% in *V*_*charge*_ compared to the dense BCZT harvester, see Figures 10K and 11K. In contrast, the harvester with a cylinder bluff body provided a stronger mechanical input, as indicated by its twice as high *V*_*oc*_ with RMS values of 0.4–1.4 V; see Figures 10E and 11E. As a result, the use of porous BCZT led to smaller, but still significant, improvement of 129% in *V*_*charge*_ compared to the dense BCZT harvester; see Figures 10O and 11O.

(ii) Advantages of porous BCZT for capacitor charging due to increased electrical impedance

The introduction of porosity is also known to increase the internal electrical impedance (1/2*πfC*) of piezoelectric materials due to the reduction in capacitance.^33^^,^^34^ This effect is undesirable for certain applications, such as sensing, where a high internal impedance makes the material behave as a high-impedance device, requiring the use of amplifiers with high input impedance to accurately capture signals. As shown in Table 1, the porous BCZT exhibited a 48% lower capacitance, which consequently increased its optimum loading resistance to 1.9 times of the dense BCZT harvester, as seen in Figure 7. Nevertheless, the higher internal impedance of the porous BCZT harvester showed no significant impact on the capacitor charging performance, as shown in Figure 8. Notably, without the need of electrical impedance matching during capacitor charging, the porous BCZT harvester continues to achieve a maximum power output that was comparable to the optimum power output obtained for the purely resistive circuit. Surprisingly, compared to the 36% increase in optimized power output observed for the porous BCZT in a purely resistive circuit (Figure 7), this enhancement reached 89% in the time-average power of the capacitor charging test (Figure 8). These findings suggest that when a piezoelectric harvester is connected to an energy harvesting circuit to charge a capacitor as a DC power supply, the increased internal impedance of porous piezoelectric is not a critical concern for the electrical impedance matching while it benefits the time-average charging power.

(iii) Advantages of porous BCZT during flow-induced vibration by decreased stiffness and damping coefficient

The voltage and power output enhancements of 3.5- and 5.1-times, respectively (Figures 10 and 11), achieved using the porous BCZT during water flow energy harvesting, were greater than the corresponding increases in the amplitudes of the respective figures of merit, *g*_33_ and *FoM*_33_, of 2.6- and 3.5-times, respectively, as shown in Table 1. This may be due to the fact that the 9% lower damping force coefficient of the porous BCZT harvester, which interacted with the flow-induced vibration (FIV), led to an enhanced vibration amplitude and, consequently, an increased stress applied to the porous BCZT. As a result, the observed variation between the voltage and power output for the dense and porous BCZT were greater than those predicted by the *g*_33_ and *FoM*_33_ figures or merit.

### Comparison of the porous BCZT harvester to other harvesters in turbulent flow

**Figure 12:**
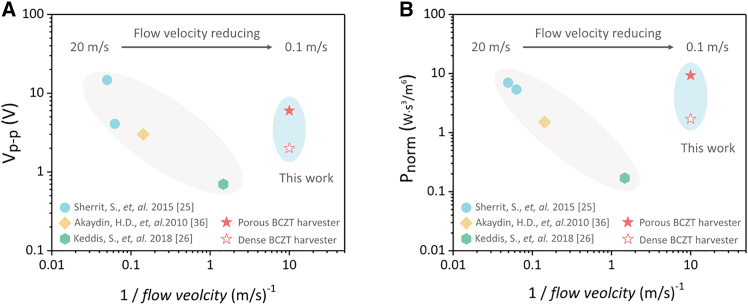
Performance comparison between BCZT harvesters in this study and the TIV harvesters reported in literature (A) peak-to-peak voltage output, and (B) normalized power output calculated via Equation 17. (TIV = turbulenced flow-induced vibration).

Based on the literature to date, the number of the studies on piezoelectric harvesters in turbulent flow has remained limited due to the randomness of the turbulence-induced vibration, which makes it difficult to simulate, predict, and optimize the oscillation behavior.^10^ As shown in Figure 12, the voltage and power output of the dense and porous BCZT harvesters developed in this paper are compared to the other reported harvesters that operate in turbulent flow. Detailed information regarding these harvesters are displayed in Table 3. Since the absolute value of the power output is correlated with the volume of the piezoelectric material (*V*_*piezo*_) and the flow velocity (*v*), a normalization approach is adopted as mentioned further^10^

(Equation 17) $$\begin{array}{c} P_{norm}=\frac{P_{out}}{V_{piezo}v^{3}} \end{array}$$

where *P*_*norm*_ is the normalized power output, enabling the comparison of piezoelectric harvester with different sizes and flow velocities.

**Table 3:** Comparison of the porous BCZT harvester to other piezoelectric harvesters in turbulent water flow (MFC = macro-fibre composites, PVDF = poly(vinylidene fluoride), ∼ = the value is estimated based on the relative data provided in the study)

Reference	Materials	Working modes	Material volume (cm^3^)	Flow velocity (m/s)	Power output	Normalized power output (W·s^3^/m^6^)	Peak-to-peak voltage output (V)	External circuits
Sherrit et al.^25^	MFC	*d*_31_	0.65	∼20	35 mW	6.7	∼15	Purely resistive
				∼16	∼15 mW	∼5.6	∼4	
Akaydin et al.^35^	PVDF	*d*_31_	0.01	7	5 μW	1.5	∼3	Purely resistive
Keddis et al.^26^	PVDF	*d*_31_	0.8	0.68	42 nW	0.17	∼0.7	Purely resistive
This work	Dense BCZT	*d*_33_	0.30	0.1	0.5 nW	1.7	1.9	Standard energy harvesting
	Porous BCZT	*d*_33_			2.8 nW	9.3	6.2	

With decreasing flow velocity, both the peak-to-peak voltage output (*V*_*p*-*p*_) and the normalized power output (*P*_*norm*_) exhibited an exponentially decreasing trend, as shown in Figures 12A and 12B, respectively, where is *x* axis is 1 / *flowvelocity*. Notably, the BCZT harvesters developed in this work deviated from these trends, addressing a key research gap in piezoelectric harvesting during low-velocity turbulent flow and demonstrating considerable voltage and power output. This enhancement can be attributed to (1) the harvester’s *d*_33_-mode of operation, and (2) the porous structure introduced into the BCZT ceramics, which improved the piezoelectric voltage constant (*g*_33_) and energy harvesting figure of merit (*FoM*_33_), as summarized in Table 1.

### Limitations of the study

This work presents the first demonstration that the use of lead-free piezoelectric porous ceramics (Ba_0.85_Ca_0.15_)(Zr_0.1_Ti_0.9_)O_3_ (BCZT), operating in *d*_33_-mode, can significantly enhance the performance of cantilever beam-based energy harvesters in turbulent, low-velocity flow conditions. Compared to the harvester utilizing dense BCZT, the harvester with porous BCZT exhibited 3.5 times higher root-mean-square value of the open-circuit voltage (*V*_*RMS*_). During a capacitor charging test, the use of porous BCZT resulted in a 2.3-times increase in the saturated charging voltage (*V*_*charge*_) reaching 2.1 V and a 5.1-times enhancement of the charging power (*P*_*charge*_), enabling DC power supply for devices requiring voltage input below 2.1 V, and successfully filling the gap of the piezoelectric harvester in low-velocity turbulent flow. These improvements were due to the higher piezoelectric voltage coefficient (*g*_33_) and piezoelectric harvesting figure-of-merit (*FoM*_33_) of the porous BCZT. Notably, the observed variations in the open-circuit voltage and power output between the dense and porous BCZT harvesters of 3.5- and 5.1-times were greater than the variations in the corresponding *g*_33_ and *FoM*_33_ of 2.6- and 3.5-times, respectively. This indicates a potential interaction between the lower damping coefficient of the porous BCZT harvester with water flow-induced vibration (FIV), leading to enhanced vibration amplitude.

In the future, further work is expected to focus on the following aspects including systematic investigation of the dependence of the porous BCZT harvester’s performance on flow velocity, modeling the solid-fluid coupling mechanism underlying turbulence-induced vibrations and optimizing the corresponding energy conversion efficiency, and exploring the mechanism responsible for the doubled voltage output observed in harvesters with electrically parallel-connected configurations.

In summary, this work demonstrates the significance of porous piezoelectric materials in water flow energy harvesting, providing a new strategy to overcome the limited performance of water flow piezoelectric harvesters in turbulent, low-velocity flow conditions, promoting the development of self-powered water monitoring systems.

## Resource availability

### Lead contact

• Requests for further information and resources should be directed to and will be fulfilled by the lead contact, Zihe Li (zl2329@bath.ac.uk).

### Materials availability

• This study did not generate new unique reagents.

### Data and code availability

• Data reported in this paper will be shared by the lead contact upon request.
• This paper does not report original code.
• Any additional information required to reanalyze the data reported in this paper is available from the lead contact upon request.

## Acknowledgments

Li acknowledges support from the NERC GW4+ Doctoral Training Partnership [NE/S007504/1] and EMD Electro-Mechanical Developments Ltd. Z.H. thanks to the support from the 10.13039/100014013UKRI Horizon Europe Guarantee funding of Marie Sktodowska-Curie Actions Postdoctoral Fellowship (grant EP/X022730/1). C.B. acknowledges support by the UKRI Frontier Research Guarantee on “Processing of Smart Porous Electro-Ceramic Transducers ProSPECT”, project no. EP/X023265/1. J.R. acknowledges support from the 10.13039/501100000266EPSRC (EP/V011332/1). For the purpose of open access, the authors have applied a Creative Commons Attribution (CC-BY) license [where permitted by 10.13039/100014013UKRI, “Open Government Licence” or ‘Creative Commons Attribution No-derivatives (CC-BY-ND) license may be stated instead] to any Author Accepted Manuscript version arising.

## Author contributions

Conceptualization, Z.L., G.H., and C.B.; methodology, Z.L., Z.H., and M.P.; investigation, Z.L. and R.Y.; writing—original draft, Z.L.; writing—review & editing, Z.L., J.R., H.K., Z.H., M.P., and C.B.; funding acquisition, Z.L., Z.H., G.H., and C.B.; resources, Z.L., J.R., H.K., R.Y., and C.B.; supervision, J.R., H.K., G.H., M.P., and C.B.

## Declaration of interests

The authors declare no conflict of interest (both financial and non-financial).

## STAR★Methods

### Key resources table

**Table:** 

REAGENT or RESOURCE	SOURCE	IDENTIFIER
**Chemicals, peptides, and recombinant proteins**		

BaCO_3_, ACS reagent, ≥99% CAS: 1314-23-4	Merck	https://www.sigmaaldrich.com/GB/en/product/sigald/237108
CaCO_3_, ACS reagent, 99.95-100.05% dry basis CAS: 471-34-1	Merck	https://www.sigmaaldrich.com/GB/en/product/sial/398101
TiO_2_, powder, 5 μm, 99% trace metals basis CAS: 1314-23-4	Merck	https://www.sigmaaldrich.com/GB/en/product/aldrich/230693
ZrO_2_, powder, 5 μm, 99% trace metals basis CAS: 1314-23-4	Merck	https://www.sigmaaldrich.com/GB/en/product/aldrich/230693

**Other**		

B2987A Electrometer / High Resistance Meter, Battery-Powered,0.01 fA	KEYSIGHT	https://www.keysight.com/hk/en/product/B2987A/electrometer-high-resistance-meter-0-0-1fa-battery.html

### Method details

#### Material manufacture

*BCZT powder*: BaCO_3_, CaCO_3_, ZrO_2_ and TiO_2_ were used as the raw materials. They were mixed according to the stoichiometric ratio of Ba_0.85_Ca_0.15_Zr_0.1_Ti_0.9_O_3_ (BCZT) and ball milled in ethanol for 48 h. The slurry was dried in an oven to remove the ethanol and then ground into a powder for calcination, where the powders were heated to 1250°C for 3 h with a heating rate of 5 °C/min. The calcined material was re-ground into powder and sieved to obtain the BCZT powders used to fabricate green bodies for the dense and porous ceramics.

*BCZT green bodies*: To fabricate green bodies for dense BCZT ceramics, the BCZT powder was mixed with a polyvinyl alcohol (PVA) solution (10 wt.% aqueous solution) as a binder, where the weight ratio of the PVA to the BCZT powders was 0.5 wt.%. The mixture was placed in a stainless-steel die and compacted with a uniaxial pressure of 80 MPa. The dense BCZT green body was dried in an oven before sintering to prevent the formation of cracks within the material caused by rapid moisture evaporation during heating. Directional freeze-casting was used to fabricate green bodies for porous BCZT ceramics, where the BCZT powders were ball milled with deionized water, PVA binder and polyacrylate dispersion agent (1 wt.% and 0.5 wt.% to the solid loading, respectively) for 48 h. The solid loading of BCZT powder in water was ∼30 vol.%. The suspension was poured into a polydimethylsiloxane (PDMS) mould with a low thermal conductivity for freezing. Aluminium tape was applied to the base of the mould surface that was in contact with the cold plate to contain the suspension and ensure a high thermal conductivity for directional freezing. To initiate freezing, an aluminium plate was cooled to -80°C to generate a temperature gradient from the bottom of the mould to the top of the slurry. After the slurry was frozen, the ice was removed by freeze drying under a vacuum for 48 h to obtain the porous BCZT green bodies.

*BCZT ceramics*: The obtained BCZT green bodies were first heated to 500°C for 2 h at a heating rate of 1°C/min to burn out the organic binder and dispersant, prior to sintering at 1400°C for 10 h with a heating rate of 5°C/min. The furnace was cooled to room temperature at a rate of 5°C/min. Their XRD patterns are shown in Figure 1A and were consistent with those of the BCZT ceramics reported in the literature.^29^^,^^30^ In the porous BCZT sample, several secondary-phase peaks were found and are marked by black triangles. These impurities may be associated with the formation of poly-titanate and CaTiO_3_ phases,^30^ resulting from A-site (i.e., Ba and Ca) sublimation during sintering and precipitation during cooling, respectively. The microstructures of the BCZT ceramics are shown in Figures 1B–1E. The dense BCZT ceramics exhibited a pore-free surface, see Figure 1B, with compact grains, as observed in the local magnification in Figure 1C, indicating that the sintering process was properly executed. Figure 1D presents the transverse-section of the freeze-casting porous BCZT parallel to the freezing direction, revealing a highly aligned lamellar porous structure was observed. Figure 1E shows the cross-section of the porous BCZT ceramics perpendicular to the freezing direction, which exhibited a lamellar structure with a higher degree of randomness in pore channel orientation.

#### Material characterisation

The porosity of the BCZT ceramics was measured by the Archimedes’ method. The XRD pattern of the BCZT ceramics were determined using X-ray diffraction (XRD) via Bruker D2 Phaser. The microstructure of the BCZT ceramics was examined by scanning electron microscopy (SEM, Hitachi SU3900). The permittivity and dielectric loss of the material were measured at room temperature using an impedance analyser (Solartron1260 Dielectric, Hampshire). The longitudinal piezoelectric charge coefficient (*d*_33_) was measured with a Berlincourt Piezometer (PM300, Piezotest). The electromechanical coupling coefficient ($k_{33}^{2}$) and mechanical quality factor (*Q*_*m*_) were measured via an impedance analyser (Agilent 4194A, Keysight), using the resonance-antiresonance method.

#### Energy harvester fabrication

*Preparation of individual BCZT samples*: The sintered BCZT ceramics were sectioned into rectangular sheets with dimensions of 15 mm in length, 10 mm in width, and 1.5 mm in thickness. To form the porous BCZT samples, the thickness direction was aligned with the freezing direction during freeze-casting. Silver epoxy was applied to the ends of the BCZT sheets (on the 10 × 1.5 mm^2^ surfaces) and cured at 120°C for 1 hour to form the electrodes. After curing the silver electrodes, the BCZT ceramics were poled using contact poling. The samples were immersed in silicone oil, heated to 60°C, and subjected to a poling field of 1 kV/mm applied parallel to the length direction of the BCZT sheet for 15 minutes. The poling field was supplied by a DC voltage source (PS/FC30P04.0-22, Glassman High Voltage, Inc.). Upon cooling to room temperature, the electric field used to achieve poling was removed. The properties of the poled BCZT sheets after 72-hour aging are given in Table 1. Notably, the porous BCZT ceramics exhibited a higher *d*_33_ compared to the dense counterpart. As reported by Li et al.,^29^ this enhancement is primarily attributed to the porous structure, which enhanced the oxidation of BCZT ceramics during sintering.

*Assembly of BCZT sheets and production of cantilever beam*: Figure 2A presents a schematic of the cantilever beam fabricated using 3D-printing, which served as the substrate for the energy harvester. The left end of the beam was fixed, while the other end was connected to a tip body subjected to external loading, thereby inducing vibrations in the beam. The poled BCZT sheets were adhered to both sides near the fixed end of the cantilever beam substrate, where the bending stress concentrated when the beam tip was vibrated. An enlarged image of the installed BCZT sheets can be seen in Figure 2B. As illustrated in Figure 2C, the two BCZT sheets had a parallel poling direction along their length direction and were designated as the ‘upper’ and ‘lower’ sheets based on their positions. Since the BCZT sheets were compressed and/or stretched along the poling direction in response to a bending stress, the harvester operated in *d*_33_-mode. The equivalent circuits of the serial and parallel electrical connections of these two BCZT sheets are illustrated in Figures 2D and 2E, respectively. The capacitance of the corresponding connection modes is shown in Figure 2F, where the serial and parallel connection configurations halve and double the capacitance of the harvester, respectively. Notably, the capacitance of individual porous BCZT sheet was 55% of the dense counterpart. Given that the mass of the porous BCZT also decreased to 53% of the dense BCZT and they shared similar geometrical dimensions, the observed difference in capacitance could be primarily attributed to the presence of porosity.

#### Energy harvesting testing

*Shaker test (dry testing)*: A shaker test was used to evaluate the harvester with a controlled mechanical load, before using it in a turbulent water environment. As shown in Figure 2G, the fixed end of the cantilever beam was connected to an electromagnetic shaker capable of providing one-dimensional reciprocating motion. The free end of the cantilever beam was equipped with a mass tip weighing approximately 27 g. The relative motion between the fixture end and the mass tip induced bending stress in the cantilever beam, resulting in a fluctuating mechanical load being applied to the harvester. Notably, despite the two BCZT sheets having the same poling direction, they consistently generated currents in opposite directions due to the opposite strain that was induced by the bending stress. The harvester’s output was measured using an electrometer (B2987A, Keysight, internal electrical impedance >1 TΩ), operating in high-impedance mode for open-circuit measurements and low-impedance mode for short-circuit measurements. The shaker test was designed to evaluate the energy harvesting performance of the harvester under idealized excitation conditions, including a constant driving voltage input of the shaker and controllable vibration frequencies.

*Water-flow-induced vibration test (wet testing)*: To characterise the water flow energy harvesting behaviour, the harvester was vertically fixed above the water flow, as shown in Figure 2H, with its free end connected to a bluff body submerged in the water flow. Vortices formed around the bluff body, thereby exerting a fluctuating force on the cantilever beam, which generated a strain in the piezoelectric material and provided the necessary energy input for the harvester, as shown in Figure 2I. The water flow channel measured 70 cm in length along the flow direction, 15 cm in height (the maximum flow depth), and 10 cm in width. The water was driven by an RS Pro 230 V Submersible Water Pump with a maximum flow rate of 216 L/min. By adjusting the flow rate of the pump, the flow velocity was maintained at approximately 0.1 m/s. The test aimed to evaluate the performance of the harvester under practical water flow conditions. No physical filter was employed to mitigate the turbulence generated by the water pump and channel walls at the inlet of the channel. The testing process can be seen in the Videos S1 and S2.

### Quantification and statistical analysis

The statistical details of the experiments are presented in Figure 3, Table 2, Figures 4, 5, and 6, and were analysed using Excel, MATLAB, and Origin. The corresponding *n* value for Figure 3 and Table 2 is one, indicating that each result was obtained from a single harvester. The *n* value for Figures 4, 5, and 6 is five, meaning that each result was derived from five test periods, each lasting 1 second. The terms center and dispersion refer to the mean and standard deviation, respectively.

## References

[1] Wang X., Xiao X., Zou Z., Dong J., Qin Y., Doughty R.B., Menarguez M.A., Chen B., Wang J., Ye H. (2020). Gainers and losers of surface and terrestrial water resources in China during 1989–2016. Nat. Commun..

[2] Borrelle S.B., Ringma J., Law K.L., Monnahan C.C., Lebreton L., McGivern A., Murphy E., Jambeck J., Leonard G.H., Hilleary M.A. (2020). Predicted growth in plastic waste exceeds efforts to mitigate plastic pollution. Science.

[3] Tang W., Llort J., Weis J., Perron M.M.G., Basart S., Li Z., Sathyendranath S., Jackson T., Sanz Rodriguez E., Proemse B.C. (2021). Widespread phytoplankton blooms triggered by 2019–2020 Australian wildfires. Nature.

[4] Brittingham M.C., Maloney K.O., Farag A.M., Harper D.D., Bowen Z.H. (2014). Ecological risks of shale oil and gas development to wildlife, aquatic resources and their habitats. Environ. Sci. Technol..

[5] Nižetić S., Šolić P., López-de-Ipiña González-de-Artaza D., Patrono L. (2020). Internet of Things (IoT): Opportunities, issues and challenges towards a smart and sustainable future. J. Clean. Prod..

[6] de Camargo E.T., Spanhol F.A., Slongo J.S., da Silva M.V.R., Pazinato J., de Lima Lobo A.V., Coutinho F.R., Pfrimer F.W.D., Lindino C.A., Oyamada M.S., Martins L.D. (2023). Low-cost water quality sensors for IoT: A systematic review. Sensors.

[7] Pagano A., Garlisi D., Tinnirello I., Giuliano F., Garbo G., Falco M., Cuomo F. (2025). A survey on massive IoT for water distribution systems: Challenges, simulation tools, and guidelines for large-scale deployment. Ad Hoc Netw..

[8] Essamlali I., Nhaila H., El Khaili M. (2024). Advances in machine learning and IoT for water quality monitoring: A comprehensive review. Heliyon.

[9] Bowen C.R., Kim H.A., Weaver P.M., Dunn S. (2014).

[10] Li Z., Roscow J., Khanbareh H., Haswell G., Bowen C. (2024). Energy Harvesting from Water Flow by Using Piezoelectric Materials. Adv. Energy Sustain. Res..

[11] Sun W., Zhao D., Tan T., Yan Z., Guo P., Luo X. (2019). Low velocity water flow energy harvesting using vortex induced vibration and galloping. Appl. Energy.

[12] Cao D., Ding X., Guo X., Yao M. (2021). Design, simulation and experiment for a vortex-induced vibration energy harvester for low-velocity water flow. Int. J. of Precis. Eng. Manuf. Green. Tech..

[13] Cao D., Ding X., Guo X., Yao M. (2021). Improved flow-induced vibration energy harvester by using magnetic force: An experimental study. Int. J. of Precis. Eng. Manuf. Green. Tech..

[14] Franca M.J., Valero D., Liu X., Jack J., Shroder F. (2022). Treatise on Geomorphology.

[15] Keddis S., Schwesinger N. (2016). Active and Passive Smart Structures and Integrated Systems 2016 (SPIE).

[16] Yadav D., Yadav J., Vashistha R., Goyal D.P., Chhabra D. (2021). Modeling and simulation of an open channel PEHF system for efficient PVDF energy harvesting. Mech. Adv. Mater. Struct..

[17] Zhao D., Zhou J., Tan T., Yan Z., Sun W., Yin J., Zhang W. (2021). Hydrokinetic piezoelectric energy harvesting by wake induced vibration. Energy.

[18] Tan D., Wang Y.-C., Kohtanen E., Erturk A. (2021). Trout-like multifunctional piezoelectric robotic fish and energy harvester. Bioinspir. Biomim..

[19] Song R., Hou C., Yang C., Yang X., Guo Q., Shan X. (2021). Modeling, validation, and performance of two tandem cylinder piezoelectric energy harvesters in water flow. Micromachines.

[20] Lakshman Rao P., Sree Sai Prasad B., Sharma A., Khatua K.K. (2022). Experimental and numerical analysis of velocity distribution in a compound meandering channel with double layered rigid vegetated flood plains. Flow Meas. Instrum..

[21] Mertes L.A.K. (1994). Rates of flood-plain sedimentation on the central Amazon River. Geol..

[22] Li T., Lee P.S. (2022). Piezoelectric energy harvesting technology: from materials, structures, to applications. Small Struct..

[23] Jadhav T., Chole A.M., Deshmukh B.T. (2017). 2017 2nd IEEE International Conference on Recent Trends in Electronics, Information & Communication Technology (RTEICT) (IEEE).

[24] Peters C., Ortmanns M., Manoli Y. (2008). 2008 51st Midwest Symposium on Circuits and Systems.

[25] Sherrit S., Lee H.J., Walkemeyer P., Winn T., Tosi L.P., Colonius T. (2015). Sensors and Smart Structures Technologies for Civil, Mechanical, and Aerospace Systems 2015 (SPIE).

[26] Keddis S., Mitry R., Schwesinger N. (2018). Active and Passive Smart Structures and Integrated Systems XII (SPIE).

[27] Li Z., Roscow J., Khanbareh H., Taylor J., Haswell G., Bowen C. (2023). A comprehensive energy flow model for piezoelectric energy harvesters: understanding the relationships between material properties and power output. Mater. Today Energy.

[28] Rawana P. (2012). https://www.gov.uk/government/publications/restriction-of-hazardous-substances-rohs-regulations.

[29] Li Z., Roscow J., Khanbareh H., Davies P.R., Han G., Qin J., Haswell G., Wolverson D., Bowen C. (2024). Porous Structure Enhances the Longitudinal Piezoelectric Coefficient and Electromechanical Coupling Coefficient of Lead-Free (Ba_0.85_Ca_0.15_)(Zr_0.1_Ti_0.9_) O_3_. Adv. Sci..

[30] Roscow J.I., Pearce H., Khanbareh H., Kar-Narayan S., Bowen C.R. (2019). Modified energy harvesting figures of merit for stress-and strain-driven piezoelectric systems. Eur. Phys. J. Spec. Top..

[31] Ottman G.K., Hofmann H.F., Bhatt A.C., Lesieutre G.A. (2002). Adaptive piezoelectric energy harvesting circuit for wireless remote power supply. IEEE Trans. Power Electron..

[32] Kraśny M.J., Bowen C.R., Michel C., Taylor J.T. (2020). Transient Analysis of a Current-Driven Full Wave AC/DC Converter for Indirect Characterization of Piezoelectric Devices during Energy Harvesting. Energy Tech..

[33] Narayan B., Li Z., Wang B., Haugen A.B., Hall D., Khanbareh H., Roscow J. (2024). Temperature-Dependent Ferroelectric Properties and Aging Behavior of Freeze-Cast Bismuth Ferrite–Barium Titanate Ceramics. ACS Appl. Mater. Interfaces.

[34] Wang Q., Li Z., Bowen C., Courtney C., Pan M., Xu Q., Chen W., Fieldhouse S., Wan C. (2024). Enhanced buoyancy and propulsion in 3D printed swimming micro-robots based on a hydrophobic nano-fibrillated cellulose aerogel and porous lead-free piezoelectric ceramics. Nano Energy.

[35] Akaydin H.D., Elvin N., Andreopoulos Y. (2010). Energy harvesting from highly unsteady fluid flows using piezoelectric materials. J. Intell. Mater. Syst. Struct..

